# Case Report: Exome and RNA Sequencing Identify a Novel *de novo* Missense Variant in HNRNPK in a Chinese Patient With Au-Kline Syndrome

**DOI:** 10.3389/fgene.2022.853028

**Published:** 2022-03-29

**Authors:** Xin Pan, Sihan Liu, Li Liu, Xu Zhang, Hong Yao, Bo Tan

**Affiliations:** ^1^ Department of Gynecology and Obstetrics, The Second Affiliated Hospital of Chongqing Medical University, Chongqing, China; ^2^ Institute of Rare Diseases, West China Hospital of Sichuan University, Chengdu, China

**Keywords:** missense variant, Au-Kline syndrome, RNA-seq, hnRNPK, clinical diagnosis

## Abstract

Au-Kline syndrome is a severe multisystemic syndrome characterized by several congenital defects, including intellectual disability. Loss-of-function and missense variants in the *HNRNPK* gene are associated with a range of dysmorphic features. This report describes an eleven-year-old Chinese boy with intellectual disability and developmental delays. Family-based whole-exome and Sanger sequencing identified a *de novo* missense variant in *HNRNPK* (NM_002140.3: c.143T > A, p. Leu48Val). In silico analysis predicted that this variant would be damaged in a highly conserved residue in the K homology 1 (KH1) domain. Bioinformatic analysis showed that the affinity change (ΔΔG) caused by this variant was -0.033 kcal/mol, indicating that it would have reduced affinity for RNA binding. Transcript analysis of the peripheral blood from this case found 42 aberrantly expressed and 86 aberrantly spliced genes (*p*-value <0.01). Functional enrichment analysis confirmed that the biological functions of these genes, including protein binding and transcriptional regulation, are associated with *HNRNPK*. In summary, this study identifies the first Chinese patient with a novel *de novo* heterozygous *HNRNPK* gene variant that contributes to Au-Kline syndrome and expands current knowledge of the clinical spectrum of *HNRNPK* variants.

## Introduction

Au-Kline syndrome (AKS) was first described in 2015 in two unrelated boys who presented a wide spectrum of abnormalities, including atypical facial features, developmental delays, and hypotonia with intellectual disability. AKS-associated facial features include long faces, ptosis, cleft palate, and oligodontia. Genetic alterations of the heterogeneous nuclear ribonucleoprotein K (*HNRNPK*) gene are responsible for the development of AKS.

*HNRNPK* is a member of the RNA-binding protein family and is involved in both physiological and pathological processes, including spermatogenesis, nervous system and ovary development, erythroid differentiation, organogenesis, and carcinogenesis (Barboro et al., 2014; Gallardo et al., 2016; Geuens et al., 2016). *HNRNPK* contains three repeat K homology domains, KH1, KH2, and KH3, which recognize target RNAs and play a central role in regulating gene expression, chromatin structure, and other genetic functions. To date, the genotypes and detailed clinical features of over 30 AKS patients have been well-characterized (Dentici et al., 2018; Gillentine et al., 2021). However, there are no reports of Chinese cases of AKS. In addition, more cases are needed to better understand the relationship between AKS and its associated pathogenic variants.

This study describes the clinical and molecular characteristics of the first Chinese AKS patient who had a novel *de novo* missense variant of *HNRNPK* (NM_002140.3: c.143T > A) and expands the current understanding of the genotypic spectrum of AKS.

### Case Presentation

**FIGURE 1:**
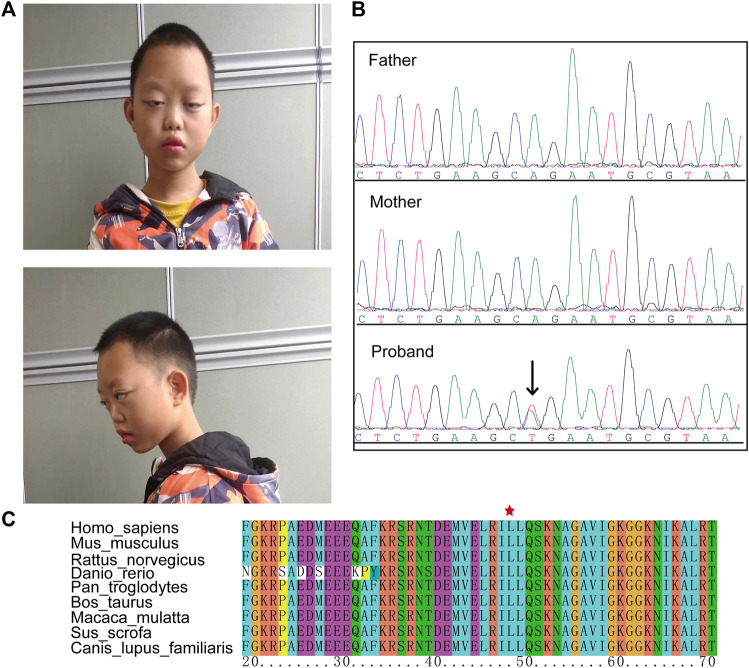
The clinical phenotype of the case. **(A)** Dysmorphic features including a long face, long palpebral fissures, ptosis, and hypoplastic alar nasi. **(B)** Sanger sequencing confirmed a *de novo HNRNPK* variant (NM_002140.3: c.143T > A) in the proband. **(C)** This variant caused an amino acid change (p. L48G) that in a highly conserved region.

The proband, a boy 11 years and 10 months of age, was the first child of nonconsanguineous Chinese parents. Prenatal ultrasounds were normal. The proband failed to raise his head and exhibited hypotonia at 6 months of age and a language delay with his first words spoken at 3 years of age. He had a moderate degree of intellectual disability. Dysmorphic features included a long face, long palpebral fissures, ptosis, and hypoplastic alar nasi (Figure 1A).

An ultrasound confirmed mild hydronephrosis and cryptorchidism at 11 years of age (Supplementary Figure S1), and oligodontia was determined by panoramic radiographs. The proband’s hearing was normal, and no significant abnormalities were found by brain magnetic resonance imaging (MRI). Chromosomal karyotype and microarray analysis results were normal.

## Methods

### Ethical Compliance

Informed consent was obtained from the patient’s parents. This study was approved by the ethics committee from the Second Affiliated Hospital of Chongqing Medical University.

### DNA Extraction, Whole-Exome Sequencing, and Variant Analysis

Genomic DNA was isolated from each participant’s peripheral blood using a blood genomic DNA extraction kit (Tiangen Biotech, Beijing, China) according to the manufacturer’s protocol. The gDNA was fragmented, captured, and sequenced using the MGI-2000 sequencing system (BGI China).

Raw sequencing reads were filtered to obtain clean reads using Fastp,(Chen et al., 2018) and FastQC (Trivedi et al., 2014) was used to evaluate the quality of sequencing data in each sample. Clean DNA sequencing reads were mapped to the human reference genome hg19 (GRCh37) using the BWA-MEM algorithm (Li and Durbin, 2009), and ambiguously mapped reads (MAPQ <10) and duplicated reads were removed using SAMtools (Li et al., 2009) and PicardTools [http://broadinstitute.github.io/picard/], respectively. SNPs and small insertions and deletions (INDEL) were identified according to the Genome Analysis Toolkit software’s best practices and variants were annotated using the Ensembl Variant Effect Predictor (VEP) (McLaren et al., 2016). According to guidelines from the American College of Medical Genetics and Genomics and the Association for Molecular Pathology (ACMG) (Richards et al., 2015), variants were classified as pathogenic (P), likely pathogenic (LP), benign (B), likely benign (LB), or variants of uncertain significance (VUS). Variant validation was performed using Sanger sequencing (ABI 3730xl Genetic Analyzer).

### RNA Sequencing and Data Preprocessing

Total RNA was isolated from peripheral blood and enriched by oligo-dT bead capture and cDNA was synthesized according to the manufacturer’s protocol. cDNA libraries were constructed using the Illumina trueSeq stranded mRNA sample prep kit protocol (Illumina). Pooled samples were sequenced using a NovaSeq 6000 sequencing system.

Raw sequencing reads were filtered to obtain clean reads using Fastp, and FastQC was used to evaluate the quality of sequencing data based on several measures, including sequence quality per base, sequence duplication level, and quality score distribution for each sample. The average quality score for overall RNA sequences was >30, indicating that a large percentage of the sequences were high quality. The clean RNA-sequencing reads were mapped to the human reference genome (hg19) using STAR (2.4.2a) with the Gencode v19 annotation (Dobin et al., 2013).

### Identification of Aberrant Gene Expression and Pathway Enrichment Analysis

Aberrant gene expression, splicing, and monoallelic expression were detected using DROP (Yepez et al., 2021) with the default filter parameters. To increase the power to detect aberrantly expressed genes, in-house data with the same sequencing and analysis pipeline was included. Genes were defined as having aberrant expression, splicing, or monoallelic expression with a *p*-value <0.01. Functional enrichment of the aberrant genes was performed with KOBAS-i, a service that provides comprehensive pathway enrichment analysis using several databases, including GO, KEGG, Reactome, and GWAS catalogs (Bu et al., 2021). An adjusted *p*-value <0.05 was selected as the threshold for significant pathways.

## Results

After trio whole-exome sequencing (Trio-WES) was performed on each family member, the causal variants were evaluated using ACMG guidelines. Results identified a novel missense *HNRNPK* variant (NM_002140.3: c.143T > A) in the patient that was absent in the parents (Figure 1B). This variant was classified as LP with the following evidence (PS2_Moderate + PM2 +PP3). This variant was not reported in the dbSNP and gnomAD databases, and predicted as pathogenic using in silico prediction tools (SIFT = 0.001, Polyphen2 = 0.99, MutationTaster = 1, and EVE = 0.945) (Frazer et al., 2021). The *HNRNPK* variant caused a missense substitution (p. Leu48Gln) localized in the K homology domains of a highly conserved region, suggesting that the variant may disrupt the binding ability of the HNRNPK protein (Figure 1C).

To prove this hypothesis, we predicted the effect of this missense variant on protein affinity with mCSM-NA (Pires and Ascher, 2017) using the PDB (Protein Data Bank) file provided by AlphaFold. The predicted affinity change (ΔΔG) was -0.033 kcal/mol, indicating that HNRNPK had reduced affinity for RNA binding. In addition, the predicted stability effect of HNRNPK was -3.541 kcal/mol, indicating that the protein was destabilized. These results suggest that the *de novo* variants (NM_002140.3:c.143T > A) may lead to loss of function of HNRNPK.

**FIGURE 2:**
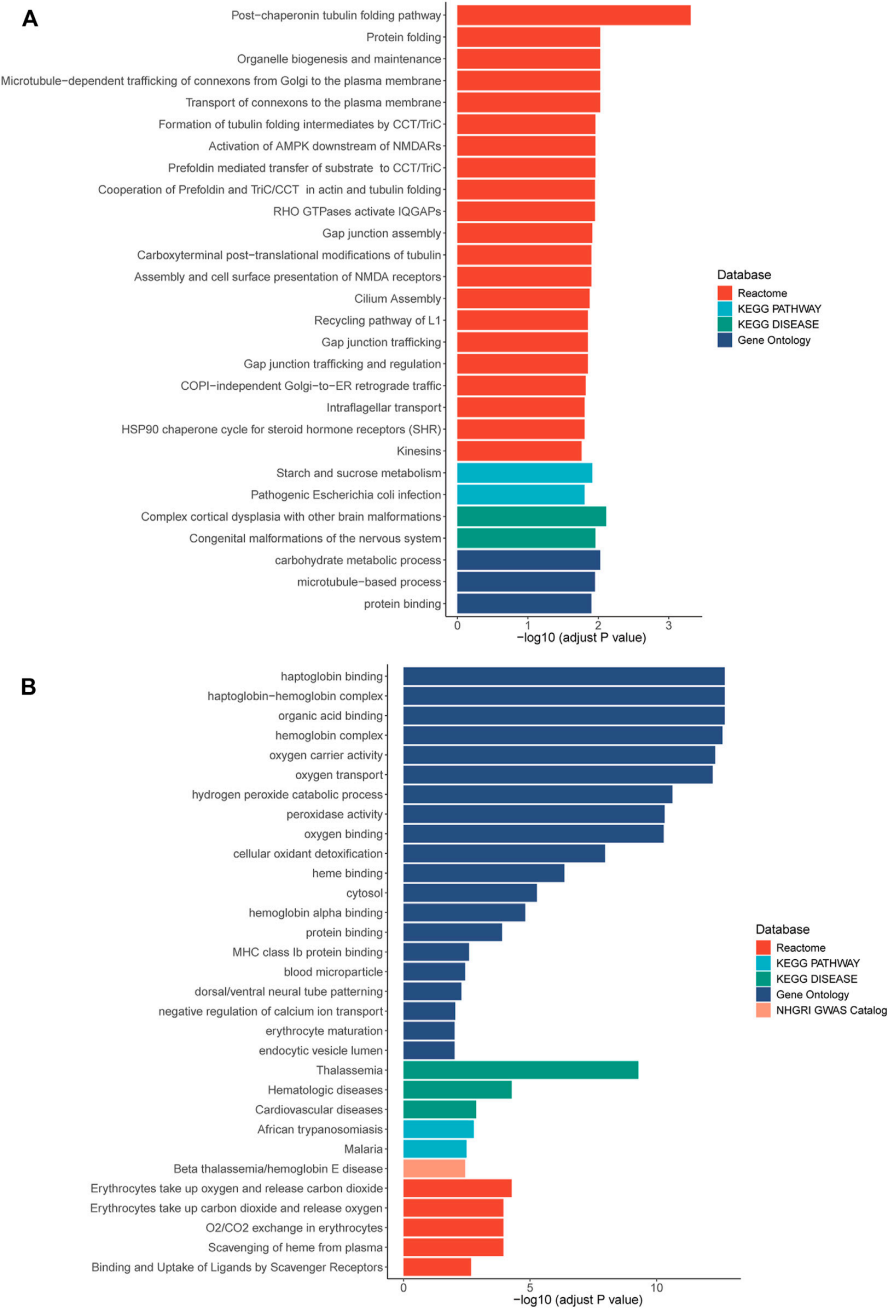
Pathway enrichment results of genes with aberrant expression **(A)** and with aberrant splicing **(B)**. Colors stand for pathways items from different database. The *X* axis shows adjusted *p*-value with log transformed. Pathways with an adjusted *p*-value <0.05 was selected as significant pathways and plotted.

RNA sequencing was also performed and 42 and 86 genes with aberrant expression and splicing were identified, respectively (Supplementary Figures S2–S5, Supplementary Tables S1, S2). While the *HNRNPK* gene was not differentially expressed, two target genes regulated by *HNRNPK*, *TUBB2A* (*p* = 0.0093) and *TUBB2B* (*p* = 0.0092), were differentially expressed. Pathway enrichment analysis showed that the biological functions of these genes correlated with protein binding, transcriptional regulation, and nervous system regulation (Figure 2; Supplementary Tables S3, S4).

## Discussion and Conclusion

In this study, we reported a patient with AKS who had multiple system anomalies, including developmental delay, facial dysmorphism, and kidney and genital abnormalities. Exome sequencing and Sanger validation showed that these phenotypes may be explained by a novel *de novo* missense variant of the *HNRNPK* gene (NM_002140.3: c.143T > A). In addition, there is a missense variant (NM_002140.3:c.142C > G; p. Leu48Val) interpreted as uncertain significance in ClinVar database which affect the same site with different amino acid.

**FIGURE 3:**
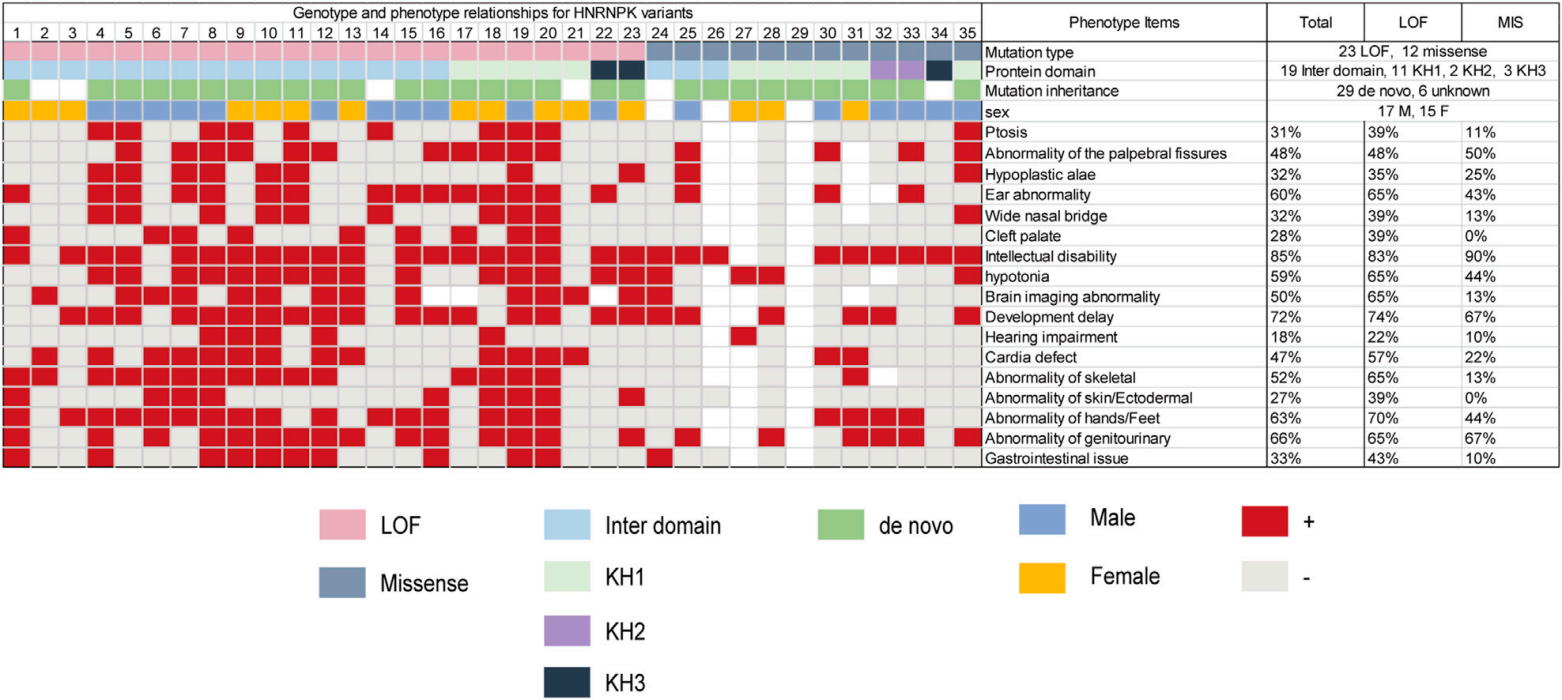
Genotype and phenotype relationships for *HNRNPK* variants. Pink boxes represent loss-of-function variants; Navy blue boxes represent missense variants; Green boxes represent *de novo*; blue boxes represent male; yellow boxes represent female; red boxes represent present. Light gray boxes represent absent; white boxes represent unreported. Total (%) represents the number of patients who were reported to have the specific phenotype (numerator) divided by the number of patients. LOF (%) represents the number of patients with LOF variants who were reported to have the specific phenotype (numerator) divided by the number of patients with LOF variants. MIS (%) represents the number of patients with missense variants who were reported to have the specific phenotype (numerator) divided by the number of patients with missense variants.

With the addition of our patient, a total of 33 pathogenic *HNRNPK* variants (21 LOF and 12 missense) that caused AKS have been reported in 35 patients (Table 1) (Miyake et al., 2017; Au et al., 2018; Gillentine et al., 2021). Three-quarters (75%) of the missense variants occurred within the KH domain (42% in the KH1 domain). In contrast, most LOF variants was in the interdomain. The most common phenotype in patients with AKS includes intellectual disability (85%), developmental delay (72%), genitourinary abnormalities (66%), and hand and foot abnormalities (63%). Phenotypic differences between individuals with loss-of-function and missense variants were observed in ptosis, wide nasal bridge, brain imaging abnormalities, cardiac defects, and skeletal and gastrointestinal abnormalities. The patient reported here had a unique phenotype of craniofacial features and oligodontia (Figure 3; Supplementary Table S5).

**TABLE 1:** Pathogenic variants identified in *HNRNPK* gene (NM_002140.3).

Patient index	Genomic (hg38)	cDNAchange	AAchange	Function	References
1	chr9:g.86592674_86592675insC	c.85_86insG	p.Glu29Glyfs^a^	LOF	Wang et al. (2020)
2	chr9:g.86592661delA	c.99delT	p.Phe33Leufs^a^25	LOF	Gillentine et al. (2021)
3	chr9:g.83973901C > T	c.402+1G > A	N/A	LOF	Gillentine et al. (2021)
4	chr9:g.83972056dupC	c.779dupG	p.Asp262^a^	LOF	Au et al. (2018)
5	chr9:g.83971976G > A	c.859C > T	p.Arg287^a^	LOF	Au et al. (2018)
6	chr9:g.83971903_83971904insAA	c.931_932insTT	p.Pro311Leufs^a^40	LOF	Gillentine et al. (2021)
7	chr9:g.83971903_83971904insAA	c.931_932insTT	p.Pro311Leufs^a^40	LOF	Gillentine et al. (2021)
8	chr9:g.83971881dupC	c.953+1dupG	p.Gly319Alafs^a^6	LOF	Au et al. (2018)
9	chr9:g.83971682dupT	c.998dupA	p.Tyr333^a^	LOF	Dentici et al. (2018)
10	chr9:g.83971671C > T	c.1008+1G > A	N/A	LOF	Au et al. (2018)
11	chr9:g.83971356del	c.1009del	p.Val337Leufs^a^13	LOF	Au et al. (2018)
12	chr9: g.83970911delC	c.1094delG	p.Gly365Valfs^a^28	LOF	Au et al. (2018)
13	chr9:g.83970896C > A	c.1108+1G > T	N/A	LOF	Gillentine et al. (2021)
14	chr9:g.83970832A > G	c.1109-13T > C	N/A	LOF	Gillentine et al. (2021)
15	chr9:g.83970161C > T	c.1361+1G > A	N/A	LOF	Okamoto, (2019)
16	chr9:g.83969356A > ATTCT	c.1385_1386insAGAA	p.Phe462LfsThr^a^10	LOF	Gillentine et al. (2021)
17	chr9:g.83977061A > C	c.157-10T > G	p.52Lys_56AsninsLeuLeuGln	LOF	Yamada et al. (2020)
18	chr9:g.83975540T > C	c.214-35A > G	N/A	LOF	Murdock et al. (2021)
19	chr9:g.83975462C > T	c.257G > A	p.Arg86His? Splicing changes?	LOF	Au et al. (2018)
20	chr9:g.83975457C > T	c.257+5G > A	p.Ile87Tyrfs^a^12	LOF	Maystadt et al. (2020)
21	chr9:g.83974592G > A	c.258-3C > T	N/A	LOF	Gillentine et al. (2021)
22	chr9:g.83970334G > T	c.1192-3C > A	N/A	LOF	Gillentine et al. (2021)
23	chr9:g.83970229delC	c.1294delG	p.Asp432Ilefs^a^24	LOF	Jarvela et al. (2021)
24	chr9:g.83977780C > T	c.65G > A	p.Arg22His	missense	Farwell Hagman et al. (2017)
25	chr9:83971694G > A	c.986C > T	p.Pro329Leu	missense	Gillentine et al. (2021)
26	chr9:g.83971691C > T	c.989G > A	p.Gly330Glu	missense	Gillentine et al. (2021)
27	chr9:g.83977035A > G	c.173T > C	p.Ile58Thr	missense	Shashi et al. (2019)
28	chr9:g.83977032C > T	c.176G > A	p.Gly59Glu	missense	Wang et al. (2020)
29	chr9:g.83977009C > G	c.199G > C	p.Ala67Pro	missense	Gillentine et al. (2021)
30	chr9:g.83975466C > T	c.253G > A	p.Gly85Lys	missense	Gillentine et al. (2021)
31	chr9:g.83975466C > T	c.253G > A	p.Gly85Lys	missense	Gillentine et al. (2021)
32	chr9:g.83973359C > A	c.443G > T	p.Arg148Met	missense	Gillentine et al. (2021)
33	chr9:g.83973338A > G	c.464T > C	p.Leu155Pro	missense	Miyake et al. (2017)
34	chr9:g.83970744G > A	c.1184C > T	p.Pro395Leu	missense	Gillentine et al. (2021)
35	chr9:g.83977702A > T	c.143T > A	p. Leu48Gln	missense	This study

A total of 33 variants from 35 patients with AKS were curated from the published literature or online databases and this

Study, study, including 21 loss-of-function variants and 12 missense variants. LOF, loss-of-function variants.

a Nucleotide numbering and to indicate a translation termination (stop) codon.

We hypothesized that missense variants affect RNA binding and thus cause dysfunction of related biological pathways that lead to the development of AKS. The *de novo* missense variant identified in this study was in the KH1 domain, and the predicted affinity change (ΔΔG) showed a reduced affinity for RNA binding. Transcriptome results identifying 42 aberrantly expressed and 86 spliced genes in the patient provided additional evidence to support the hypothesis. These genes were significantly associated with protein binding, transcriptional regulation, and nervous system function. Two of the downregulated genes, *TUBB2A* (*p* = 0.0093) and *TUBB2B* (*p* = 0.0092), are known to interact with HNRNPK at the protein level (Cerami et al., 2011). Both genes encode for the tubulin protein, which plays a critical role in neuronal function, neuronal migration, and postmigration development. Prior studies have shown that reduced expression of tubulin can lead to intellectual disability, matching the phenotype of the case in this study (Breuss et al., 2017; Jimenez et al., 2019; Schmidt et al., 2021). Further experiments are needed to assess the mechanism by which HNRNPK variants impact the development of AKS.

In summary, by integrating Trio-WES and RNA-seq analyses, we were able to better understand the role of an *HNRNPK* variant in a patient with AKS. Our findings expand the current understand of the clinical spectrum of *HNRNPK* variants.

## Data Availability

The datasets for this article are not publicly available due to concerns regarding participant/patient anonymity. Requests to access the datasets should be directed to the corresponding author.
