# Supplementary material for: Case Report: Exome and RNA Sequencing Identify a Novel de novo Missense Variant in HNRNPK in a Chinese Patient With Au-Kline Syndrome
Source: Front Genet. 2022 Mar 29;13:853028. doi: 10.3389/fgene.2022.853028 (PMC9001983; doi:10.3389/fgene.2022.853028)
Supplement: Supplementary file 1 [file DataSheet2.docx]

Supplementary Material

# Supplementary Figures and Tables

## Supplementary Figures

##
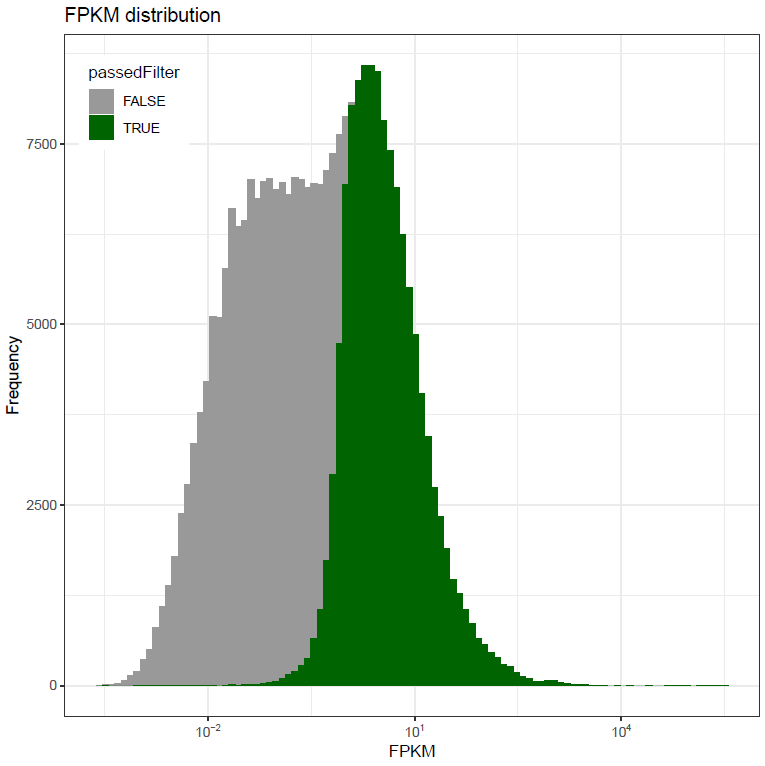


**Supplementary Figure 1.** Retaining genes with FPKM>1 in at least one sample.

(C-D) Mild hydronephrosis in the left kidney and an undescended right testicle.


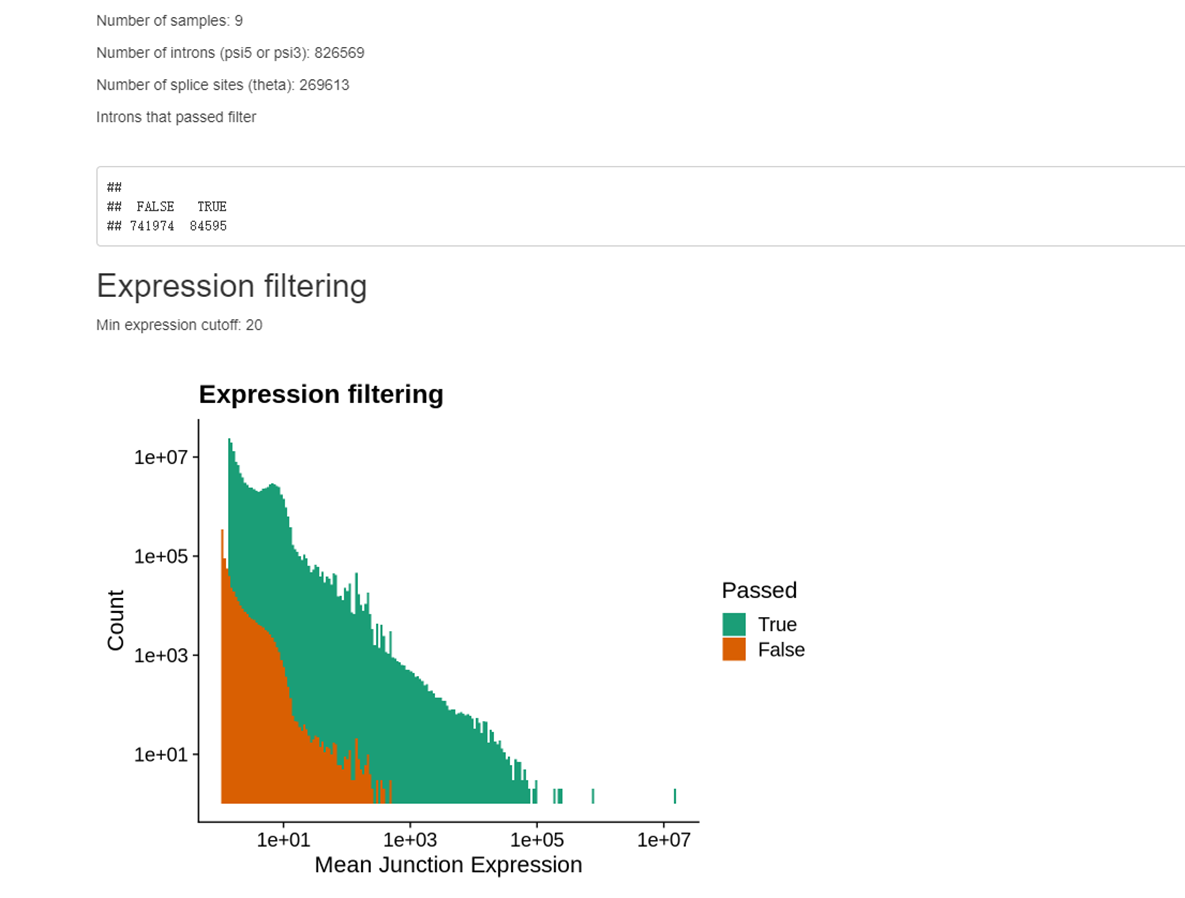
**Supplementary Figure 2:** Heatmap of gene expression before (A) and after (B) adjust confounding factors like sex and age.


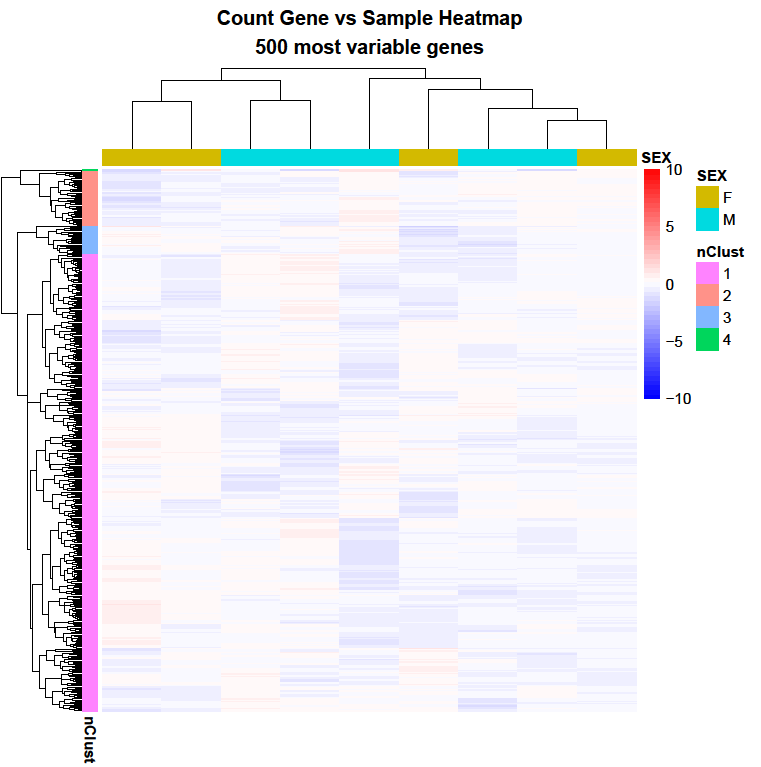

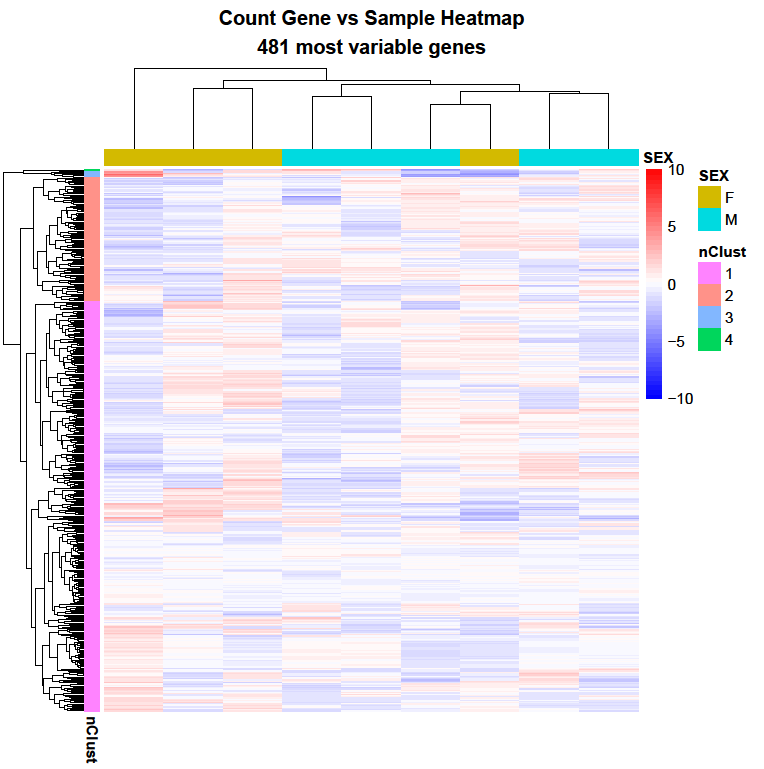


A

B

**Supplementary Figure 3.** Retaining introns with read count large than 20.


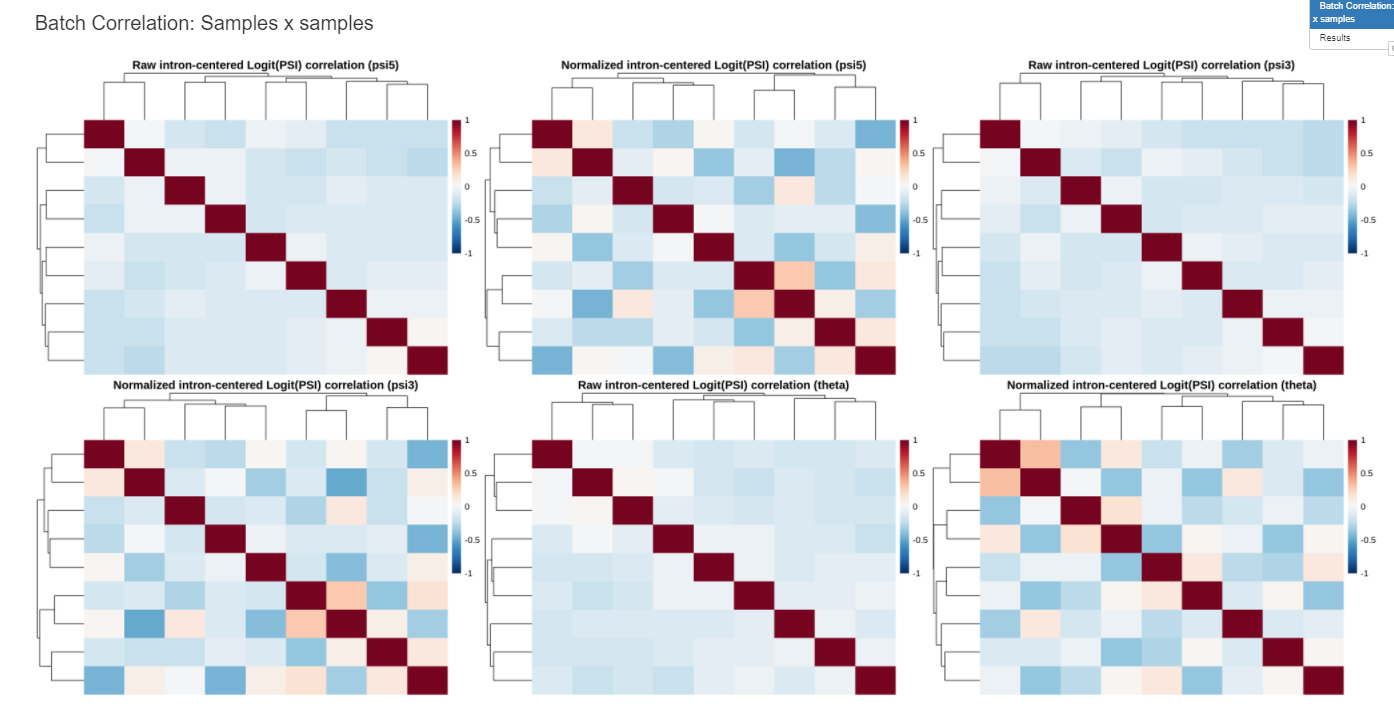


**Supplementary Figure 4.** Batch correlation for samples in aberrant splicing analysis.
